# Type III Interferons in Hepatitis C Virus Infection

**DOI:** 10.3389/fimmu.2016.00628

**Published:** 2016-12-23

**Authors:** Maude Boisvert, Naglaa H. Shoukry

**Affiliations:** ^1^Centre de Recherche du Centre Hospitalier de l’Université de Montréal (CRCHUM), Montréal, QC, Canada; ^2^Département de médecine, Université de Montréal, Montréal, QC, Canada

**Keywords:** hepatitis C, IFN-λ3, IFN-λ4, liver, SNP, HCV clearance, SVR

## Abstract

The interferon (IFN)-λ family of type III cytokines includes the closely related interleukin (IL)-28A (IFN-λ2), IL-28B (IFN-λ3), and IL-29 (IFN-λ1). They signal through the Janus kinases (JAK)-signal transducers and activators of transcription pathway and promote an antiviral state by the induction of expression of several interferon-stimulated genes (ISGs). Contrary to type I IFNs, the effect of IFN-λ cytokines is largely limited to epithelial cells due to the restricted pattern of expression of their specific receptor. Several genome-wide association studies have established a strong correlation between polymorphism in the region of IL-28B gene (encoding for IFN-λ3) and both spontaneous and therapeutic IFN-mediated clearance of hepatitis C virus (HCV) infection, but the mechanism(s) underlying this enhanced viral clearance are not fully understood. IFN-λ3 directly inhibits HCV replication, and *in vitro* studies suggest that polymorphism in the IFN-λ3 and its recently identified overlapping IFN-λ4 govern the pattern of ISGs induced upon HCV infection of hepatocytes. IFN-λ can also be produced by dendritic cells, and apart from its antiviral action on hepatocytes, it can regulate the inflammatory response of monocytes/macrophages, thus acting at the interface between innate and adaptive immunity. Here, we review the current state of knowledge about the role of IFN-λ cytokines in mediating and regulating the immune response during acute and chronic HCV infections.

## Introduction

The interferon (IFN)-λ family of cytokines was first described in 2003 by two independent groups (1, 2). By using computational analysis of unknown genes potentially corresponding to cytokines that were related to interleukin (IL)-10 and type I IFNs, Sheppard et al. identified three new cytokines, IL-28A, IL-28B, and IL-29 (2). Expression of these three cytokines could be induced in peripheral blood mononuclear cells (PBMCs) and other cell types upon poly I:C stimulation or viral infection. Furthermore, these cytokines demonstrated antiviral activity and were shown to bind to a new receptor, IL-28Rα, that forms a heterodimer with IL-10R2. Around the same time, Kotenko et al. also identified three new genes related to the IFN type I and IL-10 families (1). The new cytokines were named IFN-λ1, IFN-λ2, and IFN-λ3 (equivalent to IL-29, IL-28A, and IL-28B, respectively). The newly described cytokines were shown to bind to a new receptor complex composed of IFN-λR1 (equivalent to IL-28Rα) and the IL-10R2, signal through the Janus kinases-signal transducers and activators of transcription (Jak-STAT) pathway, and exhibit antiviral activities *via* the induction of interferon-stimulated genes (ISGs) and upregulation of major histocompatibility complex (MHC) class I. In 2013, a dinucleotide frameshift variant rs368234815 (previously termed ss469415590) (TT or ΔG) was identified in the IFN-λ region. This frameshift variant was shown to create a novel gene, IFNL4, encoding the IFN-λ4 protein (3). This new protein was related to IFN-λ3 (29.1% identity and 40.8% similarity between both proteins). Expression of IFN-λ4 activated the Jak-STAT pathway and resulted in the expression of ISGs (3). In this article, we will use the nomenclature of IFN-λ genes, protein, and polymorphisms according to the Human Genome Organization Gene Nomenclature Committee. Alternative names for IFN-λ genes and proteins (including IFN-λ specific receptor) are listed in Table 1.

**Table 1:** **Type III IFN genes and proteins**.

	Gene	Alternate gene names	Protein	Alternate protein names
Receptor	*IFNLR1*	*IL-28RA, IL-28R1, IFNLR*	IFN-λR1	IL-28RA, IL-28Rα, IL-28R1
Cytokines	*IFNL1*	*IL-29*	IFN-λ1	IL-29
	*IFNL2*	*IL-28A*	IFN-λ2	IL-28A
	*IFNL3*	*IL-28B*	IFN-λ3	IL-28B
	*IFNL4*	*–*	IFN-λ4	–

## Tissue Tropism of Type I Versus Type III IFNs

Type I and type III IFNs are related and may act in parallel *via* the same pathways. Type I IFNs (IFN α/β) can act on multiple cell types and tissues because their specific receptors (IFNAR1 and IFNAR2) are ubiquitously expressed. In contrast, IFN-λR1 expression is rather restricted and as such it affects a much more limited set of cells and exhibits reduced side effects (4). IFN-λR1 is mostly expressed by cells of epithelial origin including hepatocytes (5, 6). However, its expression on hematopoietic cells remains controversial. This issue is discussed in more detail below, but it is generally believed that the main immune cells expressing IFN-λR1 are dendritic cells (DCs) (4, 7, 8). Most studies assessed the expression of IFN-λR1 by polymerase chain reaction (PCR), evaluating the mRNA level, which might not accurately reflect expression of the protein on cell surface. It was demonstrated that immune cells [B cells, T cells, and natural killer (NK) cells] express mostly a shorter splice variant of IFN-λR1 that can be secreted (9). This secreted form could bind IFN-λ with moderate affinity and inhibit its effects. This could explain at least in part why immune cells express IFN-λR1 mRNA but lack responsiveness to IFN-λ treatment.

## Association of Type III IFN Polymorphisms with HCV Spontaneous Clearance and Response to IFN Therapy

Hepatitis C virus (HCV) infection is a global health problem. Only 25% of individuals acutely infected with HCV are able to eliminate the virus spontaneously, while the majority (~75%) develops persistent infection and chronic liver disease including fibrosis, cirrhosis, and liver cancer (10). Until 2011, the only available treatment for HCV was a combination of ribavirin and pegylated IFN-α (11). This non-specific treatment was modestly effective, especially in individuals infected with genotype 1, resulting in ~50% sustained virological response (SVR) rate defined as undetectable viral load 24 weeks following the end of treatment (12). Furthermore, the course of treatment was long (48 weeks) and associated with multiple side effects, thus significantly impacting the quality of life of the patients (13). Factors associated with higher odds of spontaneous resolution or response to IFN therapy include virus genotype, gender, and ethnicity, suggesting that genetic factors are key determinants of viral clearance (14, 15). Individuals of European ethnicities were more likely to achieve SVR compared to individuals of African ancestry (14, 16). These differences accompanied by the difficulties and side effects associated with IFN treatment prompted research into genetic factors that can predict SVR. Several genome-wide association studies demonstrated a link between single-nucleotide polymorphisms (SNP) near the *IFNL3* gene encoding IFN-λ3 and HCV infection outcome and response to treatment. These major polymorphisms are listed in Table 2. Ge et al. demonstrated that the IFN-λ3 rs12979860 SNP predicted the response to IFN treatment in an American cohort composed of multiple ethnicities infected with HCV genotype 1 (17). The favorable allele (CC genotype) was not only overrepresented in the treatment responder group but was also more prevalent in the European population compared to the African population where the unfavorable TT genotype was more prevalent. Moreover, the IFN-λ3 rs12979860 genotype was a better predictor of treatment outcome than ethnicity, since African Americans with the CC genotype were more likely to achieve SVR than the European American bearing the TT genotype (17). That study also demonstrated that the CC genotype was associated with higher baseline viral loads in all groups tested. Two other studies confirmed the same association with polymorphism in the IFN-λ3 region in Australian (18) and Japanese cohorts (19) and identified an additional SNP (rs8099917). This SNP was associated with HCV genotype 1 treatment response in the Australian cohort and confirmed with other cohorts (18). This study also used quantitative reverse transcription PCR to demonstrate that healthy individuals carrying the favorable allele (TT) expressed higher levels of IFN-λ2 and IFN-λ3 transcripts in peripheral blood. In the Japanese cohort, both SNPs (rs12979860 and rs8099917) were associated with treatment response (19).

**Table 2:** **Type III IFN gene polymorphisms**.

SNP	Common name	Alternative names	Favorable allele	Unfavorable allele
rs12979860	IFN-λ3	IL-28B	CC	TT
		IFNL4 rs12979860		
rs8099917	IFN-λ3	IL-28B	TT	GG
rs368234815	IFN-λ4	ss469415590	TT	ΔG
rs117648444	IFN-λ4-P70S		AA (IFN-λ4-S70)	GG (IFN-λ4-P70)

The favorable rs12979860 CC genotype was also associated with spontaneous clearance in untreated individuals from six different cohorts (20). In this study, Thomas et al. also observed that the C allele was more represented in Europeans compared to African individuals. More importantly, they demonstrated that the C allele was associated with spontaneous resolution of HCV infection in both ethnic groups. Moreover, the protective effect appeared to be recessive, since there was no difference between heterozygous individuals bearing the CT genotype and homozygous individuals bearing the TT genotype. This study also genotyped >2,000 individuals worldwide and demonstrated that the C allele was most prevalent in East Asia, whereas the T allele was most prevalent in Africa and an intermediate pattern with both alleles was observed in Europe. Similar results were obtained by Rauch et al. who sequenced the IFN-λ3 rs8099917 SNP and showed association of the unfavorable allele with establishment of a chronic infection and treatment failure in HCV monoinfected and HCV/HIV coinfected individuals (21). Finally, the IFN-λ3 rs12979860 SNP was also associated with spontaneous clearance and jaundice in a single-source cohort (22). The German anti-D cohort consists of 2,867 women who were exposed to HCV genotype 1b after treatment with anti-D immunoglobulin. Fifty-two percent of infected women achieved spontaneous clearance. This cohort enabled the evaluation of the role of IFN-λ3 polymorphism in spontaneous clearance without the confounding effect of virus genetics. In this cohort, it was possible to analyze genetic factors associated with spontaneous clearance in 190 women. Results demonstrated that spontaneous clearance was strongly associated with the IL-28B/IFN-λ3 genotype (22). The highest rate of clearance was observed in women homozygous for the favorable C allele (CC, 64.2% clearance), the lowest rate of clearance was observed in women homozygous for the unfavorable T allele (TT, 6.1% clearance), and intermediate levels of clearance were observed in heterozygous women (CT, 24.4% clearance) (22). IFN-λ3 favorable genotype was also associated with clearance upon reinfection in high-risk people who inject drugs (23).

In 2013, a new dinucleotide polymorphism rs368234815 (previously termed ss469415590) located near the *IFNL3* gene was identified, and the variants TT or ΔG were associated with a frame shift resulting in either production of a new protein, IFN-λ4, (ΔG) or absence of the protein due to the introduction of a frameshift creating an early stop codon (TT) (3, 24). This new polymorphism was in high linkage disequilibrium with the IFN-λ3 rs12979860 polymorphism and was found to be a stronger predictor of HCV spontaneous resolution and treatment outcome of chronic HCV (3, 25, 26). Another group reported association of the TT/ΔG polymorphism with HCV treatment outcome in a large European cohort (27). Given that the IFN-λ3 rs12979860 was located within the newly discovered IFN-λ4 region, it was suggested to change its nomenclature to IFN-λ4 rs12979860 (24).

## Mechanisms Underlying the Role of IFN-λ Polymorphisms in HCV Clearance

The exact mechanisms underlying the role of IFN-λ polymorphisms in HCV clearance are not well understood. It was proposed that such polymorphisms may influence the expression of IFN-λ cytokines during HCV infection and their downstream effects on expression of ISGs and innate and adaptive immune cells. Although it was demonstrated early on that IFN-λ SNPs may influence expression of the IFN-λ transcripts in PBMCs (18), data evaluating the circulating levels of IFN-λ cytokines during acute and chronic HCV were inconclusive. Data from the chimp model of HCV infection demonstrated that type III IFNs were strongly induced upon HCV infection at the gene and protein level and correlated with ISG expression and viral load (28). In humans, while some studies associated the favorable IFN-λ3 CC allele with higher serum levels of IFN-λ (29), others demonstrated the reverse correlation (7). One report also found no difference in serum levels of IFN-λ between HCV treatment responders and non-responders (30). Our group has demonstrated that serum levels of IFN-λ3 were highly variable, but were lower in individuals bearing the favorable IFN-λ3 CC allele (31). Altogether, type III IFN genotyping has been, so far, a more accurate predictor for HCV infection or treatment outcome compared to the circulating levels of the cytokines.

How the expression of IFN-λ4 would interfere with HCV clearance or treatment response is not fully understood. It was shown that the protein is only poorly secreted (3, 32). Nevertheless, the protein could interact with the same receptor as IFN-λ3 (IFN-λR1 and IL-10R2) and displayed similar levels of activation of ISGs and antiviral activity (32). It remains possible that IFN-λ4 has other functions apart from activation of ISGs, perhaps through the interaction with an intracellular receptor. Both IFN-λ3 and IFN-λ4 polymorphisms were associated with the level of expression of the type I IFN receptor *IFNAR1* in PMBCs (33). Individuals carrying both favorable alleles expressed the highest level of *IFNAR1*, while individuals bearing both unfavorable alleles exhibited lower levels. Treatment of PBMC with IFN-α confirmed that individuals with both favorable alleles and the highest *IFNAR1* expression also exhibited the highest ISG induction.

Several early studies demonstrated a link between the liver expression levels of ISGs before IFN treatment initiation and treatment outcome (34–37). A higher level of expression of a set of ISGs and genes involved in IFN regulatory pathways (ISG15 and USP18) was observed in non-responder patients before treatment and predicted treatment outcome (34). Furthermore, the level of expression of several ISGs was shown to correlate with IFN-λ genotypes, with the unfavorable alleles associated with higher hepatic levels of ISGs (37, 38). Unphosphorylated IFN-stimulated gene factor 3 (ISGF3) is induced by type III IFNs and sustains expression of USP18, a negative regulator of IFN signaling, resulting in unresponsiveness to IFN-α treatment (39). Comparison of levels of ISGs before and after treatment further demonstrated that the high basal expression levels in non-responders did not increase above pretreatment level, whereas there was a strong ISGs induction in the SVR group (40). This suggests that the baseline high ISG levels in non-responders render them unresponsive to further IFN stimulation upon therapy. Extending this hypothesis to explain why the expression of IFN-λ4 would be detrimental for HCV infection and treatment outcome, patients expressing a less active variant of the IFN-λ4 protein had better odds of achieving spontaneous clearance or SVR (41). This study demonstrated that the IFN-λ4-S70 protein (SNP rs117648444) exhibited reduced ISG activation and antiviral activity *in vitro*. When comparing infection and treatment outcome in a large cohort, individuals bearing genetic variants resulting in no IFN-λ4 production had the highest odds of clearance/SVR, followed by those expressing the IFN-λ4-S70 impaired protein and finally those expressing the IFN-λ4-P70 fully active protein had the lowest odds of achieving clearance/SVR.

**Figure 1:**
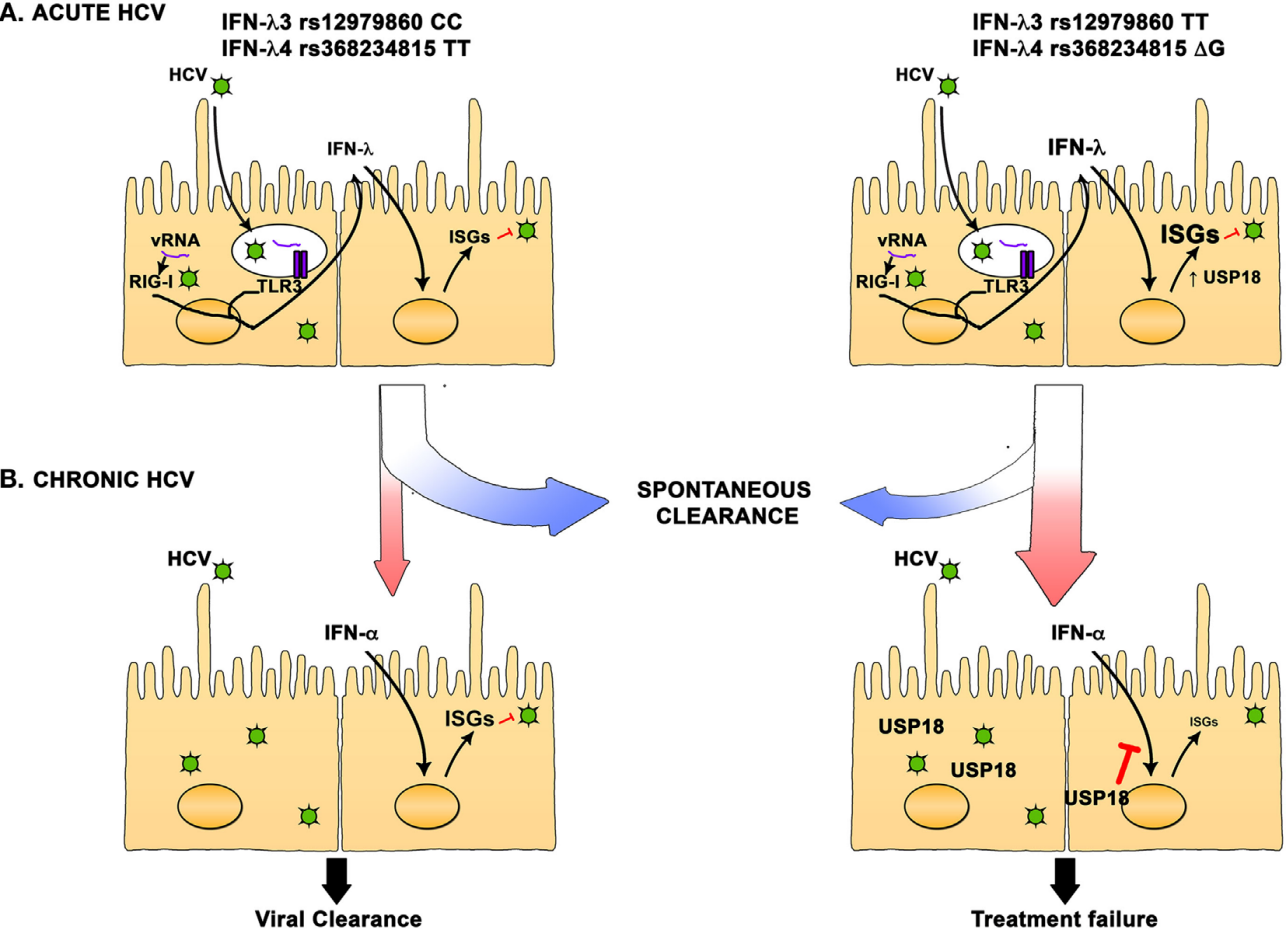
**Interferon (IFN)-λ polymorphisms are associated with hepatitis C virus (HCV) spontaneous resolution and response to treatment**. **(A)** In HCV acutely infected individuals, IFN-λ3 rs12979860 CC and IFN-λ4 rs368234815 TT favorable alleles are strongly associated with spontaneous resolution. Hepatocyte infection triggers the production of IFN-λ that then induce the expression of hundreds of interferon-stimulated genes (ISGs). In individuals carrying the favorable alleles, production of IFN-λ is moderate, and the ISGs induction is more focused, leading to an effective antiviral state and increased rate of spontaneous resolution of infection. On the other hand, carriers of the unfavorable alleles exhibit stronger IFN-λ production and a more diverse array of ISGs can be detected. This will also induce the expression of USP18, an inhibitor of the IFN signaling pathway, leading to an impaired antiviral state and an increased rate of chronic HCV infection. **(B)** In chronically infected individuals carrying the favorable allele, basal level of IFN-λ and ISGs is relatively low and treatment with IFN-α can induce a potent antiviral state leading to viral clearance or SVR. In individuals carrying the unfavorable allele, a high basal level of IFN-λ, ISGs, and USP18 will lead to a refractory state and unresponsiveness to the IFN-α treatment and failure to clear the infection.

On the basis of current knowledge, we can elaborate a model where during acute HCV infection, innate immune responses are induced in hepatocytes that trigger the production of type III cytokines that stimulate a variety of antiviral ISGs. In individuals carrying the favorable IFN-λ3 rs12979860 CC and IFN-λ4 rs368234815 TT alleles, production of IFN-λ is controlled, and the induction of ISGs is more focused leading to an effective antiviral state and increased rate of spontaneous resolution of infection. In contrast, carriers of the unfavorable alleles exhibit stronger IFN-λ production and a more diverse array of ISGs. This will also induce the expression of USP18, an inhibitor of the IFN signaling pathway, leading to an impaired antiviral state and to an increased propensity to develop chronic infection. This effect is not absolute, and some individuals carrying the favorable alleles will develop chronic infection. In chronically infected individuals carrying the favorable allele, basal levels of IFN-λ and ISGs will be relatively low and treatment with IFN-α can induce a potent antiviral state leading to viral clearance or SVR. In individuals carrying the unfavorable allele, a high basal level of IFN-λ, ISGs, and USP18 will lead to a refractory state and unresponsiveness to the IFN-α treatment, and failure to respond to treatment (Figure 1).

## Type III IFNs and Innate Immunity in the Liver

HCV is a hepatotropic infection and investigation of the early steps of viral replication, and the innate immune response is hindered by the difficulty to access the infected tissue, i.e., the liver. *In vitro* systems usually show low level of viral replication, and animal models to study the immune response are limited to chimpanzees that are no longer used in research. Thus, limited information is available about the activation of the innate immune response in the liver of HCV-infected individuals during acute infection. Nevertheless, chimpanzee data have demonstrated strong induction of ISGs in the liver early after infection irrespective of the outcome toward resolution or chronicity (42, 43). Examining the kinetics of type I versus type III IFNs demonstrated that HCV-infected chimpanzees exhibited rapid induction of type III IFNs in the liver. This was associated with upregulation of ISGs but minimal induction of type I IFNs (44). Similarly, infection of primary human hepatocytes (PHHs) induced production of type III IFNs that were associated with induction of a distinct set of ISGs compared to type I IFNs (44, 45). Sheahan et al. used PHHs and laser capture microdissection to compare the transcriptional profile of HCV-infected hepatocytes to adjacent uninfected cells (46). They demonstrated that infected cells had a transcriptional profile dominated by innate immunity genes, including induction of IFN-λ genes only in infected cells. Interestingly, when comparing gene expression from donors of different IFN-λ genotypes, they demonstrated that even if a greater number of genes were induced in the unfavorable (TT) allele group, the response in the favorable allele group (CC) was more focused toward antiviral and cell death responses, and unsurprisingly, viral replication was more limited in donors bearing the favorable allele (46). Onabajo et al. also used an *in vitro* system of PHHs and hepatic cells and demonstrated that IFN-λ4, while highly retained inside cells, is also secreted and induces strong ISGs response in surrounding cells, including the expression of IP-10 (47). However, IFN-λ4 expression was associated with reduced proliferation and increased cell death (47). Ferraris et al. used PHHs of different IFN-λ3/4 genotypes to investigate the mechanisms associated with HCV clearance (48). Treatment of HCV-infected cells with either IFN-α or IFN-λ1 decreased viral load only in cultures carrying the favorable IFN-λ3/4 alleles (48). They also showed that, in both PHHs and liver biopsies of HCV-infected subjects, IFN-λ1, IFN-λ3, and ISGs production were higher in carriers of the unfavorable allele. Silencing of IFN-λ1 in unfavorable allele context restored IFN-α antiviral activity, suggesting that the high basal IFN-λ and ISG expression blocked further activation by IFN-α treatment (48). The unresponsiveness observed in the context of the unfavorable IFN-λ3/4 alleles was shown to be driven by upregulation of USP18 (39). In liver biopsies from individuals with chronic HCV, it was also shown that the favorable IFN-λ4 rs368234815 TT genotype was associated with increased degranulation capacity (CD107a+) from T, NK, and NKT cells, which correlated with serum ALT and AST levels (49). This suggests increased innate immune activation in the livers of these individuals.

Hepatocytes are not the only source of type III IFNs in the liver. Hepatic stellate cells (HSC), normally in a quiescent state, become activated following liver damage induced by HCV infection and may modulate intrahepatic immune responses. HSCs activated with the TLR-3 ligand poly I:C exhibit an antiviral effect when co-cultured with HCV-infected hepatocytes (50). Supernatants of activated HSCs demonstrated an antiviral effect that could be blocked by antibodies specific to the IL-10R2. These *in vitro* results strongly suggest that HSCs can participate in the innate immune response in the liver *via* the production of IFN-λ. Finally, DCs can also act as a key source and regulator of type III IFNs in the liver and the peripheral blood.

## IFN-λ Interaction with Hematopoietic Cells

The interaction of type III IFNs with hematopoietic cells is not fully understood. In contrast to type I IFNs whose receptors are ubiquitously expressed, type III IFNs have a limited number of target cells, because their receptor (IFN-λR1 and IL-10R2 heterodimer) expression is highly restricted to the cells of epithelial origin including hepatocytes and few hematopoietic cells (4–7). Although, IFN-λR1 transcripts could be detected in several hematopoietic cells, it has been problematic to detect its expression on cell surface. It has also been reported that hematopoietic cells may express a soluble splice variant that may influence their capacity to respond to type III IFNs (9, 51). In the following sections, we will discuss in details the effect of type III IFNs on different types of hematopoietic cells.

### Monocytes and Macrophages

It was shown that IFN-λR1 was expressed on monocyte-derived macrophages, but not monocytes (52). Monocyte-derived macrophages responded to IFN-λ1 treatment by phosphorylation of STAT-1 and increased production of cytokines such as tumor necrosis factor (TNF), IL-10, and IL-12p40 following TLR stimulation. Similar effects were observed after treatment with IFN-λ2 or IFN-λ3. Furthermore, contrary to IFN-α, IFN-λ1 enhanced cell surface expression of *IFNGR1* on monocyte-derived macrophages, thus enhancing IL-12p40 and TNF production after stimulation with IFN-γ (52). Polymorphism in the IFN-λ3 SNP rs12979860 also impacted the activation of monocytes where individuals of the TT unfavorable genotype produced lower levels of IL-12 upon activation of their monocytes with the TLR ligand R848 (53). Thus, a better IFN-λ response could potentiate the antiviral and inflammatory response of monocytes and may indirectly mediate viral clearance by boosting the induction and priming of the adaptive immune response.

### NK Cells

Cytotoxic and antiviral functions of NK cells depend on a tightly regulated balance between activation and inhibitory signals. The main inhibitory mechanism is *via* binding of the killer cell-Ig-like receptors (KIR) with MHC class I molecules (54). The polymorphism within the KIR and MHC class I genes results in interactions of different strengths and degrees of activation of NK cells that correlate with HCV infectious outcome (55). IL-28B/IFN-λ3, HLA-C, and KIR variants could additively predict response to IFN therapy in chronic HCV, suggesting a collaborative effort between type III IFNs and NK cells during viral clearance (56). Activation of NK cells, associated with the success of IFN-based treatment, was studied in relation to IFN-λ3 polymorphism, and patients carrying the unfavorable IFN-λ3 allele expressed higher levels of expression of the inhibitory receptor NKG2A on NK cells and were more likely not to respond to treatment (57). These observations further underscored the potential effect of type III IFNs on NK cells.

In the context of acute infection, *KIR2DS3* and the IFN-λ3 SNP rs12979860 unfavorable T allele synergized to increase the risk of chronic infection (58). This study also suggested a direct link between IFN-λ and NK cells, showing reduced IFN-γ production by NK cells upon IFN-λ treatment (58). However, these data were difficult to reproduce in other cohorts. The IFN-λ3 rs12979860 CC genotype was associated with decreased levels of the inhibitory receptor NKG2A after infection resolution (31). Individuals bearing the CC genotype also displayed increased NK cell function measured by IFN-γ production after stimulation irrespective of infectious outcome suggesting that IFN-λ genotype influenced NK cell function but that this was not sufficient to achieve spontaneous HCV clearance (31). Although *IFNLR1* mRNA expression could be detected in NK cells (59, 60), they express very low levels of the specific type III IFN receptor (IFN-λR1) on cell surface, even after IFN-α stimulation (31, 61, 62). Treatment of purified NK cells with IFN-λ had no effect on neither NK cytotoxicity nor cytokine production (31, 60, 62, 63). On the other hand, it was reported that the level of expression of IFN-λR1 could be upregulated by IFN-λ treatment (59) and studies in IFN-λR1^−/−^ mice have demonstrated that this receptor is required for optimal antitumoral *in vivo* activity of NK cells (64), suggesting that in some activation context, NK cells could become sensitive to type III IFNs.

Given the lack of activation of NK cells by IFN-λ (31, 60, 62, 63), indirect mechanism were investigated. IFN-λ1 affected NK cells indirectly *via* the activation of monocyte derived macrophages. Macrophages activated by IFN-λ1 produced cytokines of the IL-12 family (IL-12p40) that could then activate NK cells leading to increased IFN-γ production. This activation was determined by polymorphisms in the IFN-λ3 gene, and the presence of monocytes was essential (53). This suggests that HCV-infected individuals bearing the unfavorable IFN-λ3 allele have an impaired monocyte function. Monocytes can activate NK cells through the production of IL-12 or IL-18. Stimulated monocytes from CC genotype background produced significantly more IL-12p40 and IL-12p70 compared to monocytes of the CT or TT genotype. Blocking IL-12 and not IL-18 abolished the IFN-λ association with the level of NK cell activation by monocytes, suggesting that IL-12 is a major player in the interplay between monocyte and NK cells that is associated with IFN-λ3 genotype in HCV-infected subjects (53).

Analysis of NK cell phenotype and function in chronic HCV infection demonstrated that CD56^bright^ NK cell subsets are significantly more cytotoxic than in healthy donors based on TRAIL and CD107a expression (65). This effect was independent of the IFN-λ3 rs12979860 genotype, but subjects carrying the TT genotype exhibited the highest levels of TRAIL+ and CD107a+IFN-γ+NK cells. In the same study, CD56^dim^ NK cells of TT genotype individuals produced more TNF-α. Accordingly, individuals with the TT genotype also had a higher proportion of polyfunctional NK cells (65).

In conclusion, whether by a direct or indirect mechanism, it appears that type III IFNs can modulate NK cells activation and functions but further investigation is required to identify the exact mechanism.

### Dendritic Cells

Dendritic cells are important antigen-presenting cells and have a central role in mediating the link between the innate and adaptive immune response. DCs also are a major source of type III IFNs (8, 60, 66). Stimulation of DCs (*in vitro* and *ex vivo*) with HCV RNA induced the production of both type I and type III IFNs and the levels were associated with IFN-λ3 rs12979860 genotype, with the favorable CC allele leading to the highest IFN type III production (66). Type I and type III IFNs produced by DCs could control HCV replication *in vitro*, suggesting again an important role for type III IFN in HCV infection (66). During chronic HCV infection, serum levels of IFN-λ1 were lower compared to HCV resolvers and healthy controls (29). In acute HCV infection, IFN-λ1 serum levels were variable as described earlier (29, 31). Interestingly, HCV proteins E2 and NS3 inhibited IFN-λ1 production by stimulated DCs, suggesting that IFN-λ1 is an important immune mediator in HCV infection (29). On the other hand, treatment of DCs with IFN-λ altered their function toward a dysfunctional help to T cells (7). IFN-λ-treated DCs exhibited decreased T cell stimulation capacity by upregulating PDL1 expression. In addition, IFN-λ-treated DCs promoted the expansion of regulatory T cells (Tregs), further impeding with the immune response. Further research will be needed to clarify the role of IFN-λ and DCs during a viral infection, such as HCV.

### CD4 and CD8 T Cells

The link between IFN-λ and T cells is less studied compared to the link with cells of the innate immune response. A study by Bes et al. showed that CD4 T cell responses to HCV, assessed by IFN-γ enzyme-linked immunospot (ELISpot) assay, were of a higher frequency in the unfavorable allele (non CC) group (67). This was unexpected since a stronger immune response is normally associated with HCV spontaneous clearance. However, they tested a limited number of patient (n = 69; with 38 samples with positive ELISpot response), and there was a lot of variability between samples. In a more recent study, Scheurich et al. tested the breadth and frequency of CD4 responses to HCV and stratified their results according to the IFN-λ3 rs12979860 genotype (68). However, they did not find any difference between the various IFN-λ groups. Genetics studies showed that polymorphisms of MHC class I and MHC class II are associated with spontaneous clearance of HCV infection, independently of the IFN-λ3 polymorphism (69, 70). Protective alleles were shown to have additive effect, suggesting that innate and adaptive immunity contribute independently to the prediction of a favorable outcome following acute HCV infection. However, these independent associations do not rule out the possibility that type III IFN could modulate CD4 T cells responses. Indeed, data from other models suggest that IFN-λ can indeed impact CD4 T cells (71–73). It was reported that naive and memory CD4 T cells express IFN-λR1 mRNA and that T cell stimulation in presence of IFN-λ inhibited IL-4, IL-5, and IL-13 production, thus impeding the development of Th2 helper response without affecting cell proliferation (72, 73). IFN-λ also inhibited the upregulation of IL-4Rα on the surface of stimulated naive CD4 T cells, thus limiting the Th2 polarizing effect of IL-4 on these cells. IFN-λ-treated cells expressed significantly less GATA3, the transcription factor that is master regulator of Th2 differentiation, further supporting the hypothesis that IFN-λ has inhibitory effect on the development of a Th2 response (73). Whether such mechanisms are implicated during HCV infection remains to be seen. It is also tempting to speculate that the effect of IFN-λ polymorphism on production of IL-12 by monocytes may indirectly influence priming of the HCV-specific CD4 and CD8 T cells and the generation of antiviral Th1 responses.

### B Cells

The role of the B cells and neutralizing antibody responses during acute HCV infection remains unclear. Some studies showed that early anti-HCV antibody response is associated with higher rate of spontaneous clearance of the virus during primary infection and reinfection (74–78), but other studies showed no association (79–82). To our knowledge, no data are currently available on the role of IFN-λ polymorphisms in the B cell response against HCV but it can be inferred from other models. Generally, the role of IFN-λ on B cells was only modestly studied. One recent article by de Groen et al. demonstrated that both naive and memory B cells express IFNLR1 mRNA and that IFN-λ1 can activate B cells (83). Treatment of B cells with IFN-λ1 led to increased expression of ISGs (Mx1 and OAS1) as well as increased expression of TLR7. IFN-λ1 also enhanced IgM and IgG production from TLR7/8-stimulated B cells. Finally, IFN-λ1 stimulation increased the proliferation of B cells stimulated with TLR7/8 agonist. However, after TLR2 or TLR9 stimulation, IFN-λ1 had no effect on the antibody production or proliferation (83). Globally, this study suggests that IFN-λ1 can enhance B cell response, but only under certain stimulation conditions.

In another context, Egli et al. showed that the IFN-λ3 rs8099917 GG genotype was associated with a higher rate of seroconversion after influenza vaccination in a cohort of immune-suppressed transplant patients (84). In addition, GG genotype carriers showed lower Th1 responses after PBMC stimulation with influenza antigens, suggesting that the IFN-λ3 rs8099917 genotype can affect the Th1/Th2 balance. Accordingly, adding IFN-λ3 in the stimulation medium increased the Th1 cytokine production and reduced Th2 cytokine production (84). These observations were confirmed in a cohort of healthy volunteers where IFN-λ3 treatment enhanced Th1 cytokine profile and reduced production of Th2 cytokines after influenza stimulation (84). Also, IFN-λ3 decreased B cell proliferation and antibody production. Interestingly, it was also demonstrated that adding peptides that block the effect of IFN-λ3 during the stimulation led to an increased antibody production, suggesting that blocking IFN-λ3 during influenza vaccination could improve the seroconversion rate and thus have a better protective effect (84).

These two aforementioned studies used different type III IFNs to stimulate B cells (IFN-λ1 or IFN-λ3) as well as different antigenic stimulation. Hence, there is no clear conclusion on whether type III IFNs exert a beneficial or detrimental effect on B cell function and antibody production, and additional studies with standardized stimuli are warranted.

## Summary of the Role of Type III IFNs on Hematopoietic Cells

**Figure 2:**
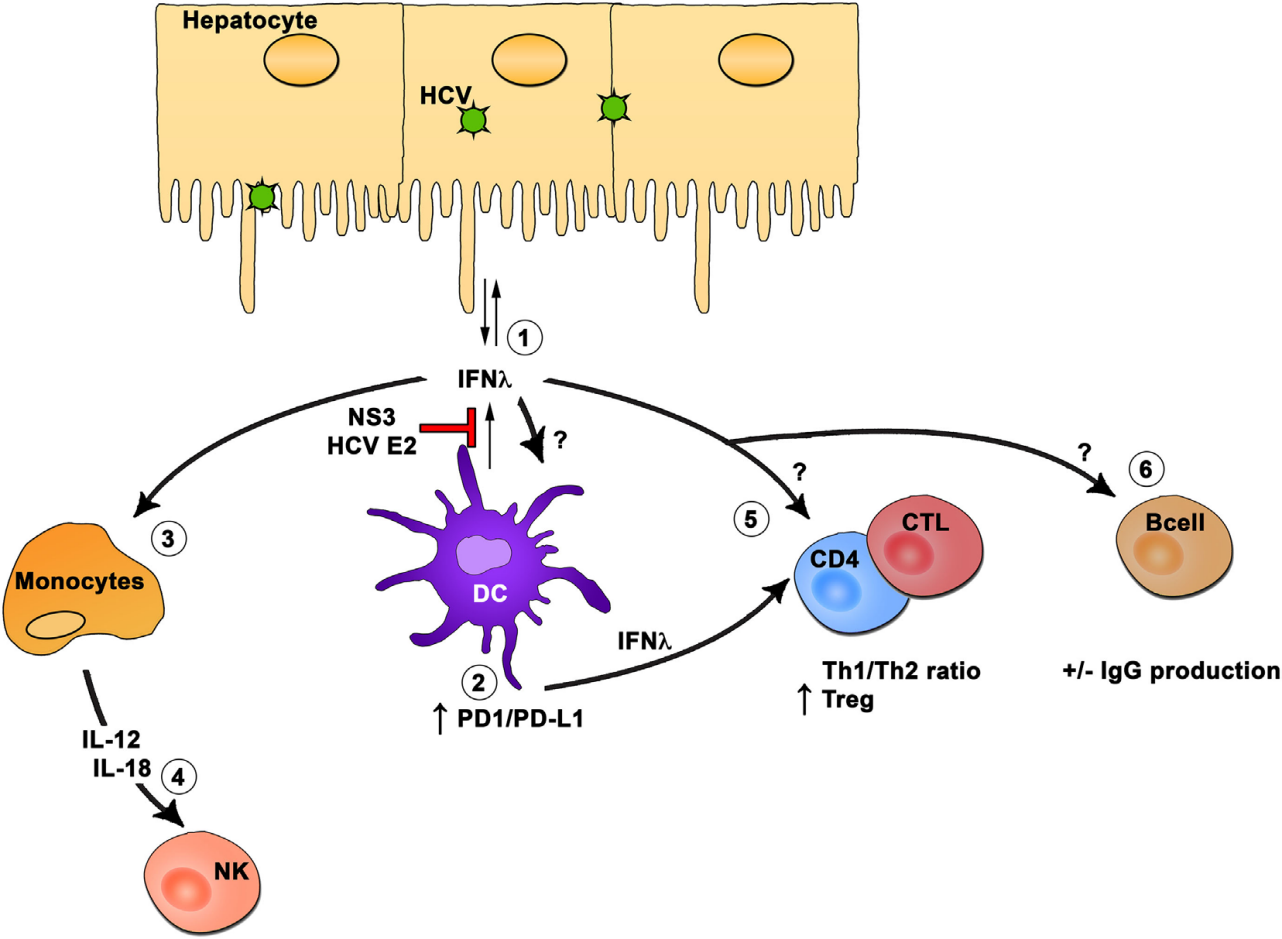
**Interferon (IFN)-λ modulation of hematopoietic cells**. In the hematopoietic compartment, dendritic cells (DCs) are the main producers of IFN-λ (1). The production of IFN-λ by DCs can inhibit hepatitis C virus (HCV) RNA replication in hepatocytes. However, HCV proteins E2 and NS3 can inhibit IFN-λ production by DCs. Hematopoietic cells express variable levels and splice variants of IFN-λR1, and conflicting results were obtained about the effect of IFN-λ treatment on these cells. HCV-exposed DCs or DC treated with IFN-λ display reduced stimulation of T cells through upregulation of PDL1 and enhanced proliferation of regulatory T cells (2). Monocytes are responsive to IFN-λ treatment resulting in interleukin (IL)-12 and IL-18 production (3). In turn, these cytokines will influence natural killer cell function (4), and thus, IFN-λ is an important component of the innate immune response to HCV. The role of IFN-λ on CD4 and CD8 T cells (5) as well as on B cells (6) in the context of HCV remains understudied, but studies suggest that IFN-λ could modulate the CD4 Th1/Th2 ratio and can also have a positive or negative impact on IgG production by B cells.

Within the hematopoietic compartment, DCs are the main producers of IFN-λ, and they can travel between the liver and peripheral blood. The production of IFN-λ by DCs can inhibit HCV RNA replication in hepatocytes. However, HCV proteins E2 and NS3 can also inhibit IFN-λ production by DCs. Hematopoietic cells express variable levels and splice variants of IFN-λR1, and conflicting results were obtained about the effect of IFN-λ treatment on these cells. HCV exposed DCs or DCs treated with IFN-λ display reduced stimulation of T cells by upregulating expression of PDL1 and enhanced proliferation of Tregs. Monocytes are responsive to IFN-λ treatment resulting in IL-12 and IL-18 production. In turn, these cytokines can influence NK cell functions, and thus, IFN-λ is an important component of the innate immune response to HCV. The role of IFN-λ on CD4 and CD8 T cells as well as on B cells in the context of HCV remains understudied, but studies suggest that IFN-λ could modulate the CD4 Th1/Th2 balance and can also have a positive or negative impact on IgG production by B cells (Figure 2).

## Type III IFNs and HCV-Specific Immunity During Pregnancy

In HCV-infected women, a sharp decrease in HCV viral load is sometimes observed after childbirth, suggestive of a boost in the immune response following delivery (85, 86). It was recently demonstrated that beside a stronger T cell response, the presence of the favorable CC IFN-λ3 rs12979860 genotype was significantly associated with this high decrease in viral load (87). Considering the high linkage disequilibrium between IFN-λ3 rs12979860 genotype and IFN-λ4 rs368234815 genotype, the IFN-λ4 was also associated with the decrease in viral load postpartum (87). It is well known that women’s immune system is altered through pregnancy to avoid a reaction against the fetus (88). It is postulated that the innate immune system will play a significant role against pathogens, while the adaptive immune responses are dampened by increased Tregs activity (89, 90). Another recent study showed that the expression of innate immunity genes is enriched in postpartum women compared to control (91). Interestingly, ISGs level in women of the CT or TT IFN-λ3 rs12979860 genotype remained elevated as late as 24 weeks after childbirth, while women with the CC IFN-λ3 genotype were comparable to non-pregnant controls (91). This reflects what was observed in the context of acute HCV where individuals with non-favorable CT or TT IFN-λ3 genotype had a higher baseline ISG expression in the liver.

## IFN-λ During Treatment with Direct-Acting Antivirals (DAA) and HCV-Related Liver Disease

With the development of novel DAAs that are highly effective (~100%) against most genotypes, treatment has switched to IFN-free regimens. Limited studies have indicated that polymorphism in the IFN-λ region may influence the response to DAAs, especially if IFN is still used in the combination (92, 93). However, given the high rate of response to DAAs and the availability of multiple products on the market, IFN-λ has lost its predictive value, and testing for it before treatment is no longer recommended except in specific situations where a DAA and IFN combination may still be warranted (94).

Chronic HCV infection is associated with an increased risk of liver-related illness, such as fibrosis, cirrhosis, and hepatocellular carcinoma (HCC). The link between IFN-λ polymorphisms and HCV-related liver disease is not completely clear and was reviewed in detail elsewhere (95). One study showed that the favorable CC IFN-λ3 rs12979860 allele, associated with better chance of HCV clearance, is also associated with higher blood ALT levels, indicative of increased liver inflammation. However, IFN-λ rs12979860 polymorphism was not associated with fibrosis progression in the same cohort (96). Another study did not find any association of the IFN-λ polymorphism with any of the observed associated liver illness (decompensated cirrhosis, HCC, liver-related death, and all-cause mortality) (97). On the other hand, Bochud et al. demonstrated that particularly in non-genotype 1 HCV-infected individuals, the favorable IFN-λ alleles were associated with increased inflammation and higher fibrosis scores (98). In agreement with this, Eslam et al. observed a significant association between IFN-λ3 rs12979860 polymorphism and liver necroinflammatory activity, serum level of AST and ALT, as well as fibrosis score and progression (99). Once again the association was stronger in individuals infected with HCV genotype 3 than those infected with HCV genotype 1. Also, in patients carrying the IFN-λ4 rs368234815 unfavorable allele (ΔG), there was a correlation between the frequency of CD107a expressing cells and the serum ALT levels, suggestive of increased liver damage (49). In the context of HCC, two studies associated IFN-λ3 rs12979860 unfavorable CT or TT alleles with liver cirrhosis and the development of HCC in patients chronically infected with HCV (100, 101). However, this was not confirmed in two other independent studies in Japanese (102) or Italian cohorts (97). Finally, HCV-related liver disease is a multifactorial problem, and the independent association of genetic factors may not be a clear cut. Fortunately, with the development of highly effective DAAs, it is expected that these complications will be less frequent as SVR will be achievable in most patients.

## Concluding Remarks

Type III IFNs exhibit strong antiviral activity, and yet, the expression of a functional IFN-λ4 protein was strongly associated with failure to clear HCV infection either spontaneously or after IFN-based treatment. A recent study suggested that humans suppress IFN-λ4 expression through various mechanisms and hence immune functions may be dependent on other type III IFNs (103). Data accumulated so far suggest that a higher baseline ISG expression level is associated with induction of a refractory state, where further IFN treatment has no beneficial effect. With the new era of anti-HCV IFN-free DAA therapies, the role of type III IFNs during therapy has become somewhat irrelevant but its role in mediating spontaneous clearance during acute HCV infection and modulating the cross-talk between innate and adaptive immunity remains highly pertinent. This is applicable not only for HCV infection but also for other viral infections and response to vaccines. Furthermore, the recently described role of type III IFN polymorphisms in driving immunity postpartum is just the tip of the iceberg as it will become increasingly relevant to mother–infant health and vertical transmission of various pathogens.

## Author Contributions

Both the authors reviewed the literature and wrote this manuscript.

## Conflict of Interest Statement

The authors declare that the research was conducted in the absence of any commercial or financial relationships that could be construed as a potential conflict of interest.
